# Defending Against the Homodyne Detector-Blinding Attack on Continuous-Variable Quantum Key Distribution Using an Adjustable Optical Attenuator

**DOI:** 10.3390/e27060631

**Published:** 2025-06-13

**Authors:** Yijun Wang, Yanyan Li, Wenqi Jiang, Ying Guo

**Affiliations:** 1 School of Automation, Central South University, Changsha 410083, China; 2 School of Computer Science, Beijing University of Posts and Telecommunications, Beijing 100876, China

**Keywords:** homodyne detector-blinding attack, continuous-variable quantum key distribution, adjustable optical attenuator, feedback structure

## Abstract

A homodyne detector, which is also a common element in current telecommunication, is a core component of continuous-variable quantum key distribution (CV-QKD) since it is considered the simplest setup for the distinguishing of coherent states with minimum error. However, the theoretical security of CV-QKD is based on the assumption that the responses of the homodyne detector are always linear with respect to the input, which is impossible in practice. In the real world, a homodyne detector has a finite linear domain, so the linearity assumption is broken when the input is too large. Regarding this security vulnerability, the eavesdropper Eve can perform the so-called homodyne detector-blinding attack by saturating the homodyne detector and then stealing key information without being detected by the legitimate users. In this paper, we propose a countermeasure for the homodyne detector-blinding attack by using an adjustable optical attenuator with a feedback structure. Specifically, we estimate the suitable attenuation value in the data processing of CV-QKD and feed it back to the adjustable optical attenuator before the detector in real time. Numerical simulation shows that the proposed countermeasure can effectively defend against homodyne detector-blinding attacks and ensure the security of the Gaussian-modulated coherent state protocol with finite-size effect.

## 1. Introduction

Continuous variable quantum key distribution (CV-QKD) has received considerable attention because it can encode many secret bits per quantum system and is very suitable for short-range high-rate implementations [1,2,3]. Moreover, CV-QKD is typically based on so-called “coherent states” (the optical states of a good-quality laser) and can be fully implemented at room temperature by using commercial homodyne/heterodyne detectors, which have good compatibility with existing optical communication devices [4]. To date, the most powerful protocol of CV-QKD is the Gaussian-modulated coherent state (GMCS) protocol, or, equivalently, the GG02 protocol, since its theoretical security has been proven with individual attacks, collective attacks, and coherent attacks, in both asymptotic cases and finite-size regimes [5,6,7,8,9]. In addition, most experiments were performed using the GMCS protocol [10,11,12], whose data processing is analyzed with composable security [13,14,15].

However, the theoretical security of the GMCS protocol has been proven under some assumptions [1], such as that both Alice and Bob are trusted without being accessible to the eavesdropper Eve. In other words, the security of CV-QKD is broken if the real-world system does not follow these assumptions. For example, Ref. [16] shows that when some of the losses and noises of Alice or Bob are untrusted, the former security is broken and the bound of secret key rate decreases. This is an important trade-off of CV-QKD, i.e., the value of the secret key rate decreases for increasing levels of security [17]. Another important assumption is that the devices of the CV-QKD system follow their ideal models without unwanted deviation. Unfortunately, in the real world, the operation of the device may deviate from its ideal model caused by the attack of Eve, which leads to the problems of practical security. Specifically, the problems of practical security are due to the gap between the ideal model and the actual model in operation. If the problems existing in actual operation are not fully characterized, Eve can use these security vulnerabilities to support her attack so that she can steal the key information without being detected by Alice and Bob. For example, Eve can perform the laser seeding attack, which injects light into the source’s laser [18,19] and then leads to Alice and Bob making a wrong estimation on both the transmittance and excess noise, as with the secret key rate.

In addition to the source side, detectors are usually regarded as the most vulnerable part of the CV-QKD system. For example, Eve can use the device defects of Bob’s homodyne detector to perform the so-called homodyne detector-blinding attack [20], which does not deceive the shot noise calibration by changing the local oscillator (LO) like a calibration attack or a wavelength attack [21,22], but directly attacks the detector. Since the actual homodyne detector has a limited linear range, Eve introduces an additional light to make the response of the homodyne detector exceed its limited linear range, resulting in parameter estimation deviations between Alice and Bob. Note that one assumes the response of the homodyne detector is always linear with respect to the input when performing the security proof [23]. If the broken linearity is not fully characterized and compensated, Alice and Bob may not detect the existence of Eve and continue to communicate. In this case, Eve can obtain the communication information and break the practical security of the CV-QKD system. One can remove the threat of the homodyne detector-blinding attack by the measurement-device-independent (MDI) idea [24,25,26,27], which is the final solution to completely solve the side channel of the detector. However, the current distance of CV-MDI-QKD is limited, which warrants more advanced detection for the task with longer distance in the future [17,28].

In terms of the attack principle of the homodyne detector-blinding attack, in this paper, we propose a countermeasure based on adjustable optical attenuators with a feedback structure. By using an adjustable optical attenuator, the CV-QKD system can be monitored in real time. It remains effective even when Eve suddenly increases the attack intensity. In detail, we set an adjustable optical attenuator in front of the homodyne detector at Bob’s side. The real-time attenuation value of the adjustable optical attenuator is estimated based on the measurement result after the homodyne detector-blinding attack of the detector. Then, the estimated attenuation value is transmitted to the adjustable optical attenuator, so that the measurement result of the output homodyne detector can be within its finite linear domain. After this countermeasure, Alice and Bob will not wrongly estimate the excess noise and secret key rate, which ensures the security of the CV-QKD system. Numerical simulation shows the performance of the countermeasure with the GMCS protocol under finite-size effects.

The paper is organized as follows. In Section 2, we describe the steps of the GMCS CV-QKD system. In Section 3, we show the imperfection of the homodyne detector and then introduce the principle of the homodyne detector-blinding attack. The corresponding countermeasure is proposed in Section 4, which is based on an adjustable optical attenuator with a feedback structure. Numerical simulation is demonstrated in Section 4, and we conclude the paper in Section 5.

## 2. GMCS CV-QKD System

The CV-QKD system encodes the key information in the amplitude and phase of coherent states, enabling the use of homodyne or heterodyne detectors to receive and measure the arriving signal, which has good compatibility with classical optical communication systems and low detection cost. In the CV-QKD protocol, the most representative one is the GMCS protocol, and its specific implementation process is as follows:

• Alice uses a quantum random number generator to generate random numbers. The generated random numbers are encoded on the input laser so that Alice obtains the initial coherent state. The initial coherent state has its variance attenuated to the variance $V_{A}$ set by the system through the optical attenuator. The quadratures of the coherent state are the position *X* and the momentum *P*. Both *X* and *P* follow a Gaussian distribution, and their variance is $V_{A}$, and their mean is 0. Subsequently, Alice sends the prepared coherent state and the LO together to Bob’s end through the optical fiber channel.
• Bob randomly selects the measurement basis to measure the quadratures of the *N* coherent states sent by Alice. Bob creates a set of binary random sequences with *N* size, where 0 indicates the selection of *X* for measurement and 1 indicates the selection of *P* for measurement. Then, a homodyne detector is used to measure the selected quadratures to obtain the measurement results. Subsequently, Bob publicly discloses the measurement basis results, and Alice selects to retain either the quadratures *X* or *P* based on the publicly disclosed measurement basis.
• Alice and Bob select a portion of the transmitted data for the estimation of transmittance and excess noise. Based on the results of parameter estimation, they calculate the mutual information and the upper bound of the information stolen by Eve and determine whether the communication is secure based on these results. This part of the data used for parameter estimation is discarded and does not participate in the calculation process of subsequent key generation.
• When the parameter estimation indicates communication security, Alice and Bob conduct reverse reconciliation on the remaining data. Alice and Bob first discretize their respective data, and Bob publicly discloses a small portion of the bit data to Alice. Alice corrects her own data based on the error correction information sent by Bob. The error correction information is discarded and not used to generate the key. After the error correction, new data with smaller errors is obtained. Subsequently, Alice and Bob determine the hash values based on Bob’s data and the new data corrected by Alice. If the hash values overlap, the determination is successful and the protocol continues. If the hash values do not overlap, the determination fails and the protocol terminates. After successful determination, we perform confidentiality enhancement operations on the data and finally generate a security key that can be used for data encryption.

## 3. The Principle of the Homodyne Detector-Blinding Attack

### 3.1. Imperfection of Detectors

In the CV-QKD system, the performance of homodyne detectors is usually measured by electrical noise and efficiency. However, in actual situations, the imperfections of homodyne detectors such as finite linearity domain and imbalance can effect its performance and also leave security loopholes for Eve, which break the actual security of the CV-QKD system. Homodyne detectors consist of the optical part and the electrical part, where the two main defects are the imbalance in the optical part and the finite linear domain in the electrical part.

In homodyne detectors, the signal pulses and the local oscillator (LO) pulses enter the two ports of the 50:50 beam splitter for interference at Bob’s side. The two pulses output from the other two ports of the beam splitter, which are transmitted to two identical p-i-n photodiodes. The two optical pulses are converted into two photocurrents under a certain quantum efficiency. Subsequently, the two photocurrents are subtracted and amplified over a small voltage signal through an amplifier. The voltage signal is further amplified by the second-stage amplifier and detected under the analog-to-digital converter. The final generated data represents the output signal, which is proportional to the orthogonal basis of the input optical signal. The selection of orthogonal bases is determined by the relative phase between the LO pulses and the signal pulses. Due to this imperfection of the homodyne detector’s imbalance, the intensity of the LO light is not completely eliminated. This non-negligible intensity of the LO affects the output, which causes a deviation in the output signal of the homodyne detector. To quantify the influence of this imperfection on the homodyne detector, the shot noise is analyzed when there are no signal pulses but only LO pulses sent to the homodyne detector. Considering the imbalanced homodyne detector model in Ref. [29], the output state of the homodyne detector is given by

(1) $$X_{\mathrm{HD}}=\eta \left(1-2T_{\mathrm{HD}}\right)I_{\mathrm{LO}}+2\sqrt{\eta T_{\mathrm{HD}}\left(1-T_{\mathrm{HD}}\right)I_{\mathrm{LO}}}X_{0}+X_{\mathrm{ele}},$$

where η represents the detection efficiency of the homodyne detector, $1-T_{\mathrm{HD}}$ represents the total reflectance, $1-2T_{\mathrm{HD}}$ is used to describe the total imbalance factors of the homodyne detector, $I_{\mathrm{LO}}$ is the number of photons for each LO pulses, $X_{0}$ is the vacuum state, $X_{\mathrm{ele}}$ is the electrical noise with a variance of $v_{\mathrm{ele}}$, and $T_{\mathrm{HD}}$ is the total transmission efficiency including the transmission efficiency of the beam splitter, the optical loss in the fiber channel, and the efficiency of the p-i-n photodiode. The impact of the leakage of LO on the output of the homodyne detector is expressed as the first part of Equation (1), expressed as $\eta \left(1-2T_{\mathrm{HD}}\right)I_{\mathrm{LO}}$. According to the expression of $X_{\mathrm{HD}}$, its variance can be expressed as

(2) $$V_{\mathrm{HD}}=\eta^{2}{\left(1-2T_{\mathrm{HD}}\right)}^{2}f_{\mathrm{LO}}^{2}I_{\mathrm{LO}}^{2}+4\left(1-T_{\mathrm{HD}}\right)T_{\mathrm{HD}}\eta I_{\mathrm{LO}}+v_{\mathrm{ele}},$$

where $f_{\mathrm{LO}}=\sqrt{\langle I_{\mathrm{LO}}^{2}\rangle -{\langle I_{\mathrm{LO}}\rangle }^{2}}/I_{\mathrm{LO}}$ is the ratio of the fluctuation of the intensity of LO varying with the measurement time and $\langle I_{\mathrm{LO}}^{2}\rangle$ represents the noise variance caused by the fluctuation of the intensity of LO. The leakage of LO caused by the imbalance of the homodyne detector leads to offsetting the output signal and also results in LO noise in the variance of the output signal.

In addition to the imperfection of imbalance, the electronic part of the homodyne detector also has the defect of a limited range of linear detection. Beyond this linear range, it has an impact on the output result of the homodyne detector. To ensure the unconditional safety of the CV-QKD system, an important assumption during the analysis is that the orthogonal basis of the output and input after Bob’s measurement with the homodyne detector changes linearly. In practicality, as the input increases, the photocurrent also increases. When the input exceeds a certain threshold, it saturates the electronic devices, thereby causing the output signal of the homodyne detector to become saturated. The saturation of electronic devices is generally due to the saturation of amplifiers or data collectors. The inherent characteristics of amplifiers usually cause them to saturate at just a few volts. To ensure accuracy, the data collector is generally set within the range of [−1*v*, 1*v*]. In principle, data collectors can also be set as large as possible, but not infinitely large. The two p-i-n photodiodes in the homodyne detector can also saturate; however, their saturation threshold is relatively high. The total optical power of the LO and the signal pulses is much lower than photodiodes’ threshold. Therefore, the saturation of the p-i-n photodiode is generally not the cause of the detector’s saturation. Consequently, the saturation of the homodyne detector is inevitable in practicality. The saturation of homodyne detectors is represented by a simple model. The output results of the homodyne detector after Bob passes through the analog-to-digital converter can be expressed as [30]

(3) $$X_{\mathrm{HDs}}=\left\{\begin{array}{cc} S_{\mathrm{at}}, & X_{\mathrm{HD}}>S_{\mathrm{at}}\\ X_{\mathrm{HD}}, & -S_{\mathrm{at}}<X_{\mathrm{HD}}<S_{\mathrm{at}}\\ -S_{\mathrm{at}}, & X_{\mathrm{HD}}<-S_{\mathrm{at}}, \end{array}\right.$$

where $S_{\mathrm{at}}$ and $-S_{\mathrm{at}}$ represent the upper and lower bounds of the linear range, respectively. The saturation of the homodyne detector causes a change in the linear relationship between the input and output, resulting in a deviation in the parameter estimation by Alice and Bob, in which security loopholes exist.

### 3.2. The Homodyne Detector-Blinding Attack

**Figure 1:**
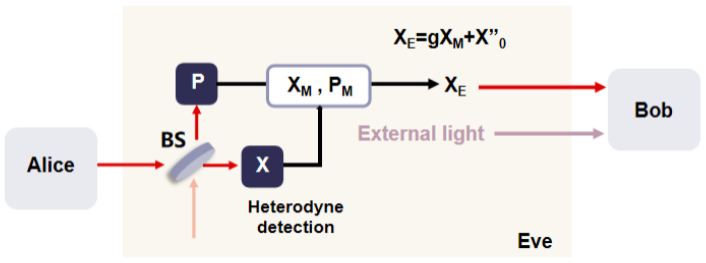
The process of the homodyne detector-blinding attack. BS: beam splitter.

Based on the above analysis of the imperfection of the homodyne detector and the principle of intercept–resend attack, Eve takes advantage of the finite-linearity domain of the homodyne detector to launch the homodyne detector-blinding attack on the system. The process of the homodyne detector-blinding attack is shown in Figure 1. Eve first intercepts all the information sent by Alice and then re-prepares and sends them to Bob. At the same time, a beam of irrelevant classical light is sent to the signal port of the homodyne detector to saturate the output signal of the homodyne detector, and communication information is obtained without being discovered by Alice and Bob.

In a homodyne detector-blinding attack, Alice prepares the Gauss-modulated coherent state $|X+iP\rangle$, where *X* and *P* are expressed as

(4) $$X=X_{A}+X_{0},$$

(5) $$P=P_{A}+P_{0},$$

where $X_{A}$ and $P_{A}$ represent the preparation variances and $X_{0}$ and $P_{0}$ represent the vacuum state with variances of one unit of shot noise. Eve intercepts the quantum signal sent by Alice and simultaneously measures the *X* and *P* with a heterodyne detector. Since *X* and *P* are symmetrical, only *X* is analyzed subsequently. The measurement result of *X* is expressed as

(6) $$X_{M}=\frac{1}{\sqrt{2}}\left(X_{A}+X_{0}+X_{0}^{\prime }\right),$$

where $X_{0}^{\prime }$ represents the vacuum state introduced by the heterodyne detector and 1/$\sqrt{2}$ is the loss caused by the heterodyne detector during the process of measurement. Based on the measured results from the heterodyne detector, Eve re-prepares the coherent state $|X_{E}+iP_{E}\rangle$ and sends it to Bob through the channel with a transmittance of *T*. The re-prepared $X_{E}$ is expressed as

(7) $$X_{E}=\frac{g}{\sqrt{2}}\left(X_{A}+X_{0}+X_{0}^{\prime }\right)+X_{0}^{\prime \prime },$$

where g=1/$\sqrt{2}$ is to compensate for the loss caused by the heterodyne detector and $X_{0}^{\prime \prime }$ is the noise with a variance of one shot noise unit introduced by Eve in re-preparing the coherent state. All the estimated parameters can be normalized to the shot noise.

As Eve re-prepares the signal pulse sent to Bob, she inserts an external laser into the signal port of Bob’s homodyne detector. The homodyne detector at Bob’s side measures the signal pulses transmitted by Eve and the external laser pulses. Ideally, the linear range of the homodyne detector is (−∞,+∞), and the output of the homodyne detector can be expressed as

(8) $$X_{\mathrm{Bi}}=\sqrt{\eta I_{\mathrm{LO}}}\left[\sqrt{\eta T}\left(X_{E}+X_{\mathrm{tech}}\right)+\sqrt{1-\eta T}X_{0}^{\prime \prime \prime }\right]+X_{E,\mathrm{ext}}+X_{\mathrm{ele}},$$

where $X_{\mathrm{tech}}$ is the technical noise generated by the devices of Eve, Alice, and Bob. $X_{0}^{\prime \prime \prime }$ is another vacuum state caused by Bob’s homodyne detector. $X_{E,\mathrm{ext}}$ are the external laser states introduced by Eve to affect the output of the homodyne detector at Bob. They can be expressed as

(9) $$X_{E,\mathrm{ext}}=\eta \left(1-2T_{\mathrm{ext}}\right)I_{\mathrm{ext}}+2\sqrt{\eta T_{\mathrm{ext}}\left(1-T_{\mathrm{ext}}\right)I_{\mathrm{ext}}}X_{0}^{\prime \prime \prime \prime },$$

where $T_{\mathrm{ext}}$ represents the overall transmission of the external laser pulse entering the signal port, $I_{\mathrm{ext}}$ is the number of photons for each external laser pulses, and $X_{0}^{\prime \prime \prime \prime }$ indicates the vacuum state that interferes with the external laser. The overall imbalance factor of the external laser pulse $1-2T_{\mathrm{ext}}$ will cause a non-negligible offset to the output results of the homodyne detector. The noise generated by the external laser inserted by Eve can be divided into two parts. One part is a kind of laser intensity fluctuation noise $V_{\mathrm{ext}}$ caused by the imbalance of the homodyne detector at Bob and the incomplete reduction in the local oscillator light. It is expressed as the first half of the expression in Equation (9). The other one is the shot noise $N_{\mathrm{ext}}$ of the external laser itself, which is expressed as the second half of the expression in Equation (9). Normalizing them into the shot noise unit $N_{0}=\eta I_{\mathrm{LO}}$, these two types of noise and the total external laser noise $V_{\mathrm{tot}}$ inserted by Eve can be expressed, respectively, as

(10) $$V_{\mathrm{ext}}=\eta f_{\mathrm{ext}}^{2}{\left(1-2T_{\mathrm{ext}}\right)}^{2}I_{\mathrm{ext}}^{2}/I_{\mathrm{LO}},$$

(11) $$N_{\mathrm{ext}}=4T_{\mathrm{ext}}\left(1-T_{\mathrm{ext}}\right)I_{\mathrm{ext}}/I_{\mathrm{LO}},$$

(12) $$V_{\mathrm{tot}}=V_{\mathrm{ext}}+N_{\mathrm{ext}}=\eta f_{\mathrm{ext}}^{2}{\left(1-2T_{\mathrm{ext}}\right)}^{2}I_{\mathrm{ext}}^{2}/I_{\mathrm{LO}}+4T_{\mathrm{ext}}\left(1-T_{\mathrm{ext}}\right)I_{\mathrm{ext}}/I_{\mathrm{LO}},$$

where $f_{\mathrm{ext}}$ is the fluctuation ratio of the intensity of external laser pulses. Since $V_{\mathrm{tot}}$ is the noise caused by the insertion of an external laser on the Bob side, the channel transmittance *T* needs to be considered when it comes to the equivalent noise on Alice’s side. The additional excess noise $\varepsilon_{\mathrm{ext}}$ caused by the external laser pulse introduced by Eve can be expressed as

(13) $$\varepsilon_{\mathrm{ext}}=V_{\mathrm{tot}}/T=\eta f_{\mathrm{ext}}^{2}{\left(1-2T_{\mathrm{ext}}\right)}^{2}I_{\mathrm{ext}}^{2}/\left(I_{\mathrm{LO}}T\right)+4T_{\mathrm{ext}}\left(1-T_{\mathrm{ext}}\right)I_{\mathrm{ext}}/\left(I_{\mathrm{LO}}T\right).$$

**Figure 2:**
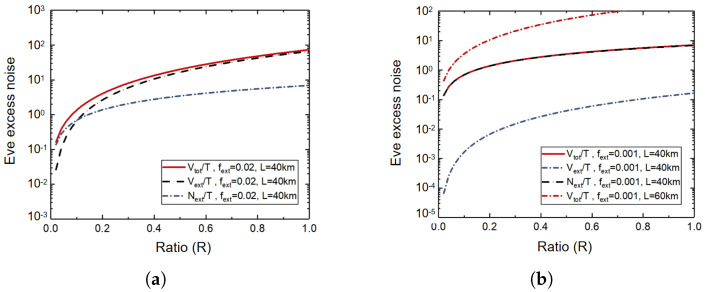
The total excess noise $V_{\mathrm{tot}}$ of the inserted external laser, the self-shot noise $N_{\mathrm{ext}}$, and the external laser intensity fluctuation noise $N_{\mathrm{ext}}$. (**a**) $f_{\mathrm{ext}}=2\%$; (**b**) $f_{\mathrm{ext}}=0.1\%$.

Figure 2 describes the total excess noise $V_{\mathrm{tot}}$, self-shot noise $N_{\mathrm{ext}}$, and external laser intensity fluctuation noise $V_{\mathrm{ext}}$ when Eve inserts an external laser under different $f_{\mathrm{ext}}$. In Figure 2, *R* is the ratio between the photon number of one of Eve’s external light pulses and one of Bob’s LO pulses, which is expressed as $R=I_{\mathrm{ext}}/I_{\mathrm{LO}}$. It can be seen from Figure 2a that when Eve uses a relatively stable laser light source, that is, $f_{\mathrm{ext}}$ = 2%, the red line and the blue line are relatively close, and as the *R* increases, the red line and the black line are closer and farther away from the blue line. At this time, the main source of the noise of the external laser pulse is its own shot noise $N_{\mathrm{ext}}$. It can be seen from Figure 2b that when Eve uses a general laser light source, $f_{\mathrm{ext}}=0.1$%, the red solid line and the black dashed line basically return, but there is a large gap from the blue dashed line. Therefore, the noise of the external laser excitation pulse mainly comes from the noise $V_{\mathrm{ext}}$ of the external laser intensity fluctuation. The cross-noise of the red dotted line at a distance of 60 km in the figure is significantly greater than that of the red solid line at a distance of 40 km. Since the channel transmittance *T* is related to the distance and *T* increases with the increase in distance, the external laser noise of Eve will also increase with the increase in the distance between Alice and Bob.

When the linear range of the homodyne detector in the CV-QKD system is infinite, Alice and Bob can easily detect the existence of Eve in the parameter estimation due to the noise introduced by the external laser pulses. However, in the actual implementation, the linear response range of the homodyne detector is finite. Eve can change the measured results of the homodyne detector by introducing external laser pulses, resulting in the output results of the homodyne detector at the Bob end exceeding their limited linear domain. When conducting parameter estimation, Alice and Bob might think that their excess noise ε is lower than the threshold for generating a zero key rate, which would make them believe that the system is secure without detecting the existence of Eve. Eve receives the classic communication between Alice and Bob to obtain the key, as both communicating parties successfully stole the information.

## 4. Countermeasure with Adjustable Optical Attenuator

**Figure 3:**
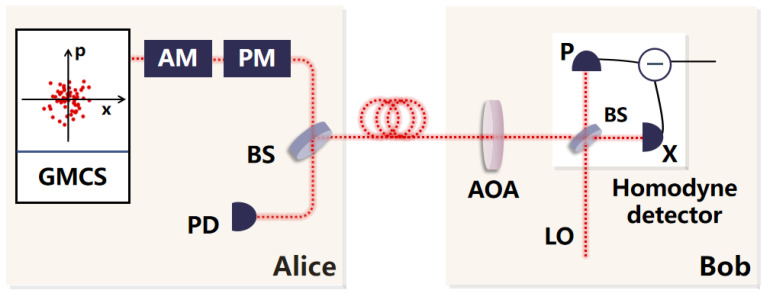
System diagram of CV-QKD based on adjustable optical attenuator. GMCS: Gaussian modulated coherent state; AM: amplitude modulator; PM: phase modulator; BS: beam splitter; PD: photodetector; AOA: adjustable optical attenuator; LO: local oscillator.

Eve exploits a homodyne detector-blinding attack to cause deviations in the estimation of estimated excess noise between the legitimate communicating parties, Alice and Bob, resulting in potential security vulnerabilities. To defend against this kind of attack, we adopt a countermeasure based on an adjustable optical attenuator, which is shown in Figure 3. When analyzing the system, we regard the adjustable optical attenuator as an ideal device and place it at the front of the homodyne detector at Bob’s side. We suppose that the initial attenuation value $\alpha_{t}$ of the adjustable optical attenuator is one. The data sent from Alice is attenuated by the adjustable optical attenuator and then enters the homodyne detector with a finite linear range for measurement. The measured results are used to estimate the attenuation value of the adjustable optical attenuator, and the generated attenuation value is fed back to the adjustable optical attenuator for superposition. Based on the attenuation value after feedback, the subsequent data sent to the Bob end is attenuated, and the process of estimating the above-mentioned attenuation value is repeated. When no attenuation value occurs, we compensate for the measured results during data post-processing. Subsequently, these data are used by Alice and Bob to estimate the excess noise and secret key rate. When there are still attenuation values generated, it continues to superimpose on the attenuation values of the original adjustable optical attenuator to attenuate the subsequent transmitted data until no attenuation values are generated. When Eve knows that we use the method of attenuating the transmitted light to defend against the attack, she increases the intensity of the external laser. The countermeasure we proposed is still effective.

We take the minimum $X_{A}$ and the minimum $X_{B}$ as the origin for establishing the coordinate system $\left(X_{A}^{\min},X_{B}^{\min}\right)$. $X_{\mathrm{sat}}^{B}$ and $-X_{\mathrm{sat}}^{B}$ represent the upper and lower bounds of the finite linear range of the homodyne detector. When Eve introduces an external laser pulse, it causes some measured results to exceed the upper bound of the finite linear range. The excess part of the measured results will be equal to $X_{\mathrm{sat}}^{B}$. In this part of the measured results, we find the minimum abscissa $X_{\min}^{A}$ and the maximum abscissa $X_{\max}^{A}$ when the measured result is $X_{\mathrm{sat}}^{B}$. Based on the points $\left(X_{A}^{\min},X_{B}^{\min}\right)$ and $\left(X_{\min}^{A},X_{\mathrm{sat}}^{B}\right)$, we can obtain the slope $k_{1}$. Based on the points $\left(X_{A}^{\min},X_{B}^{\min}\right)$ and $\left(X_{\max}^{A},X_{\mathrm{sat}}^{B}\right)$, we can obtain the slope $k_{2}$. $X_{\max}^{B}$ is the vertical coordinate when the horizontal coordinate below is $X_{\max}^{A}$ in the slope $k_{1}$. To ensure that the measurement results can be attenuated within the finite linear range of the homodyne detector, we perform to attenuate them through an adjustable optical attenuator. The attenuation value $\alpha_{t}$ can be expressed as

(14) $$\alpha_{t}=X_{\mathrm{sat}}^{B}/X_{\max}^{B},$$

After being attenuated by the adjustable optical attenuator, the measured results do not exceed the upper and lower bounds of the homodyne detector. However, measuring with the data attenuated by the adjustable optical attenuator can lead to a reduction in the measured results. The modulation variance of Alice at the transmitting end remains unchanged, while the variance of the measured result of the homodyne detector at Bob’s side will decrease, which will affect the excess noise when Alice and Bob estimate the parameters. Therefore, in the post-processing stage of the data, we compensate for the attenuated measurement results to make the estimated excess noise consistent with the results in the infinite linear domain of the homodyne detector.

## 5. Performance Analysis

Conducting a security analysis of the CV-QKD system, we first make assumptions about the implementation of CV-QKD and the external laser inserted by Eve. The simulation parameters are assumed as follows: The number of photons contained in one local oscillator’s optical pulses at Bob’s side is $I_{\mathrm{LO}}=10^{8}$. The efficiency of the homodyne detector at Bob’s side is η=0.6. The homodyne detector is a balanced homodyne detector. The LO leakage caused by its imbalance can be ignored in the study of this paper. The variance of electrical noise is $v_{\mathrm{ele}}=0.01N_{0}$. $N_{0}=\eta I_{\mathrm{LO}}$ is the shot noise variance. The upper and lower bounds of the linear detection of the homodyne detector are $|S_{\mathrm{at}}|=20\sqrt{N_{0}}$. Eve inserts an external laser beam into the signal port of Bob’s homodyne detector, and its overall transmission on Bob’s homodyne detector is $T_{\mathrm{ext}}=0.49$. The loss between Alice and Bob is $T=10^{\alpha_{l}L/10}$ with $\alpha_{l}=0.2$ dB/km; Alice’s modulation is variance $V_{A}=16$.

For the analysis of the secret key rate, we consider collective attacks and the secret key rate $K_{\mathrm{fi}}$ under the finite size, which is expressed as [6,31]

(15) $$K_{\mathrm{fi}}=\frac{n}{N}\left[\beta I_{\mathrm{AB}}-\chi_{\mathrm{BE}}-\Delta \left(n\right)\right],$$

where β=95% represents the reverse coordination efficiency, $N=10^{8}$ represents the data size of the data block, and n=0.9N represents the data block size that generates the secret key rate. $\chi_{\mathrm{BE}}$ represents the Holevo bound, which is the maximum amount of information that Eve extracts from Bob’s data. $I_{\mathrm{AB}}$ represents the Shannon mutual information between Alice and Bob, and it can be expressed as [31]

(16) $$I_{\mathrm{AB}}=\frac{1}{2}\log_{2}\frac{V_{B}}{V_{B|A}}=\frac{1}{2}\log_{2}\frac{V+\chi_{\mathrm{tot}}}{1+\chi_{\mathrm{tot}}},$$

where $V=V_{A}+1$ and $V_{A}$ represents the variance of Alice’s preparation. $\chi_{\mathrm{tot}}$ represents the total noise of the channel input, which can be expressed as

(17) $$\chi_{\mathrm{tot}}=\frac{1}{T}-1+\varepsilon +\frac{\left[\left(1-\eta \right)+v_{\mathrm{ele}}\right]}{\eta T},$$

where ε represents the excess noise estimated by Alice and Bob under the finite size. Δ(n) is related to the security of privacy amplification and is expressed as

(18) $$\Delta \left(n\right)\equiv \left(2\dim H+3\right)\sqrt{\frac{\log_{2}\left(2/\bar{\epsilon }\right)}{n}}+\frac{2}{n}\log_{2}\left(1+\epsilon_{\mathrm{PA}}\right),$$

where dimH=2 and *H* represents the Hilbert space. $\bar{\epsilon }=10^{-10}$ is the smoothing parameter, and $\epsilon_{\mathrm{PA}}$ is the probability of the privacy amplification program failing. At $n\geq 10^{4}$, Δ(n) is essentially determined by the first term of the identity and thus can be expressed as

(19) $$\Delta \left(n\right)≃7\sqrt{\frac{\log_{2}\left(2/\bar{\epsilon }\right)}{n}}.$$

**Figure 4:**
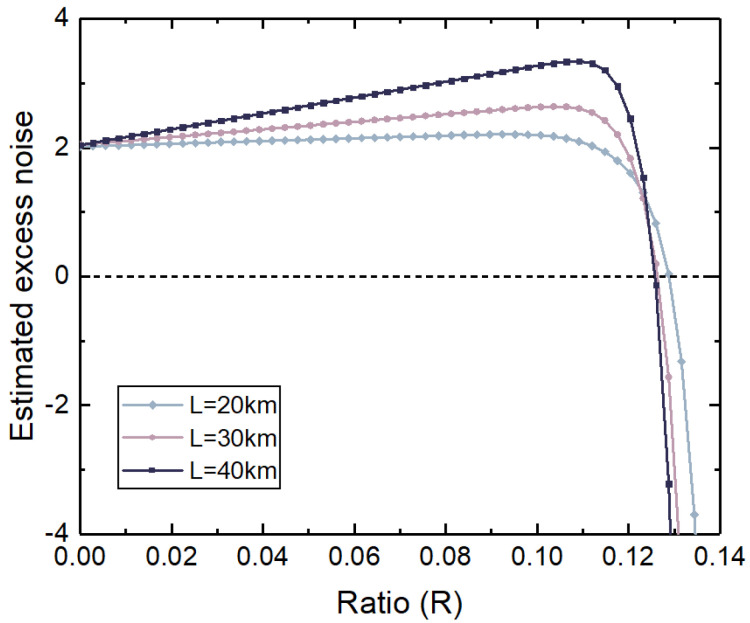
Estimated excess noise of CV-QKD system under homodyne detector-blinding attack.

Figure 4 describes the results of the estimated excess noise varying with *R* under the finite size. $V_{A}$ is the optimal Gaussian modulation variance at this distance. In the linear interval of the homodyne detector, the estimated excess noise increases with the increase in distance and *R*. At the same distance, the estimated excess noise is composed of fixed noise and variable noise that increases with *R*. The variable noise is related to the external light inserted by Eve. It can be seen from the figure that when R>0.124, the estimated excess noise decreases sharply. This is because the external beam input by Eve causes the measured results to exceed the linear measurement range of the homodyne detector, offsetting Alice and Bob’s estimation of excess noise. Eve can make the estimated excess noise close to 0 at a certain distance by choosing an appropriate *R*, that is, the generated excess noise is lower than the threshold for achieving the empty secret key rate.

**Figure 5:**
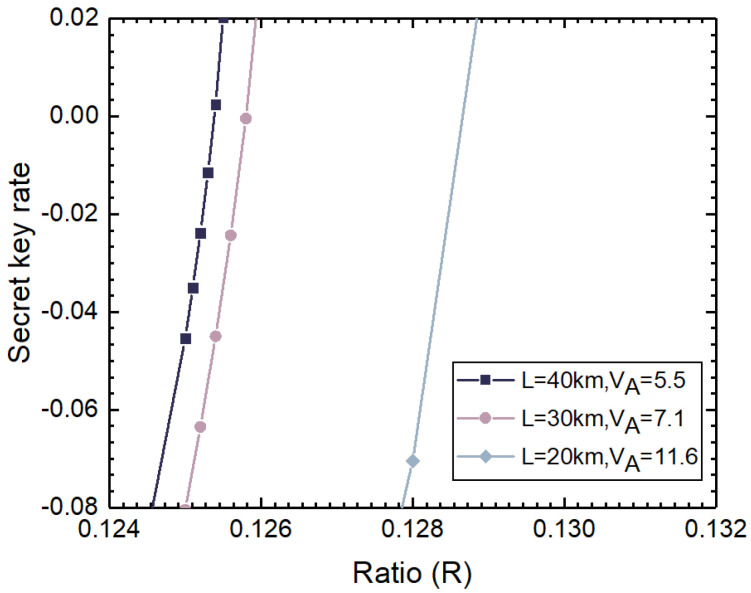
Secret key rate of CV-QKD system under homodyne detector-blinding attacks.

Figure 5 shows the estimated secret key rate corresponding to the excess noise in Figure 4. In the figure, only the part where the estimated excess noise is positive is estimated for the secret key rate, and the case where the estimated excess noise is negative is not considered. Eve can estimate $I_{\mathrm{ex}}=RI_{\mathrm{LO}}$ and $f_{\mathrm{ex}}I_{\mathrm{ex}}$ based on *R* and $I_{\mathrm{LO}}$, where $f_{\mathrm{ex}}$ represents the fluctuation ratio of the input external laser intensity. Then, we select the appropriate number of photons and the appropriate photon stability level on an external laser pulse. To generate a positive key rate between Alice and Bob, based on the parameter estimation results, the legally communicating parties believe that their communication is secure. However, due to Eve’s behavior, the generated key is not secure in practice.

**Figure 6:**
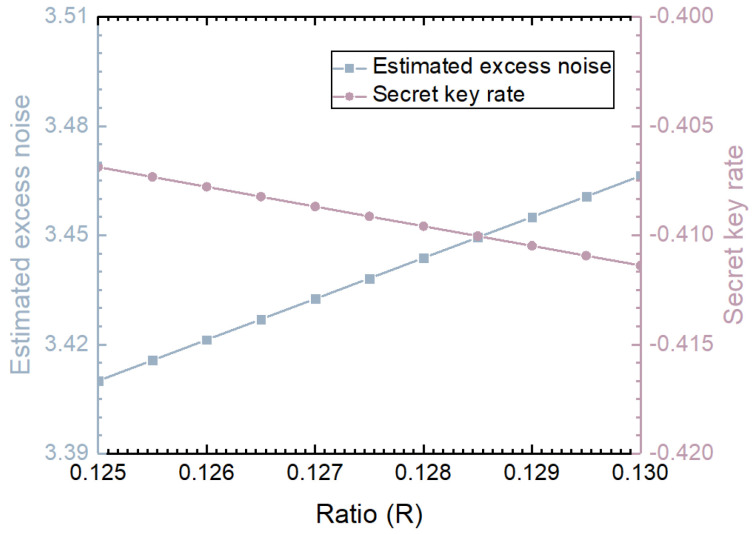
Security analysis of the CV-QKD system based on an adjustable optical attenuator defending against a homodyne detector-blinding attack.

Figure 6 shows the parameter estimation results after defending against the homodyne detector-blinding attack in the CV-QKD system based on the adjustable optical attenuator. The attenuation value is estimated based on the output results of the homodyne detector after the homodyne detector-blinding attack, and then the attenuation value is fed back to an adjustable optical attenuator to attenuate the subsequent data. Since estimating the parameters after resisting the homodyne detector-blinding attack at different distances leads to similar conclusions, we take a distance of 40 km and the Alice modulation variance $V_{A}=5.5$ as an example to describe the parameter results after the countermeasure in Figure 6. The blue line in the figure represents the estimated excess noise, and the pink line is the key rate result corresponding to the estimated excess noise of the blue line. After implementing the adjustable optical attenuator, the measured results show that within the finite linear domain of the homodyne detector, the estimated excess noise is greater than two shot noise units and keeps increasing with the increase in *R*. Under these estimated excess noises, the secret key rate is less than zero. Alice and Bob can determine that the system has been attacked based on the negative key rate, even if the communication is terminated. Therefore, the method based on the adjustable optical attenuator can effectively resist the homodyne detector-blinding attack and ensure the security of system communication.

## 6. Conclusions

In this paper, we have analyzed the homodyne detector-blinding attack under a finite size and proposed a countermeasure based on an adjustable optical attenuator with a feedback structure. By estimating the attenuation value in the data processing stage and feeding it back to the adjustable optical attenuator, the measurement result of the output homodyne detector can be within its finite linear domain. Then, Alice and Bob will not wrongly estimate the excess noise and secret key rate. Numerical simulation shows the effectiveness of the proposed countermeasure. In addition, it remains effective even when Eve suddenly increases the attack intensity or changes the wavelength of the inserted external laser pulses. According to the negative key rate, the two legally communicating parties can detect the existence of Eve and immediately terminate the communication, successfully defending against the homodyne detector-blinding attack.

## Data Availability

All data generated or analyzed during this study are included in this published article.
